# Eunicellin-Based Diterpenoids, Hirsutalins S–V, from the Formosan Soft Coral *Cladiella hirsuta*

**DOI:** 10.3390/md13052757

**Published:** 2015-04-30

**Authors:** Tzu-Zin Huang, Bo-Wei Chen, Chiung-Yao Huang, Tsong-Long Hwang, Chokkalingam Uvarani, Chang-Feng Dai, Ping-Jyun Sung, Jui-Hsin Su, Jyh-Horng Sheu

**Affiliations:** 1 Department of Marine Biotechnology and Resources, National Sun Yat-sen University, Kaohsiung 804, Taiwan; E-Mails: slime112229@gmail.com (T.-Z.H.); a6152761@yahoo.com.tw (B.-W.C.); betty8575@yahoo.com.tw (C.-Y.H.); uvaranichem@gmail.com (C.U.); 2 Graduate Institute of Natural Products, School of Traditional Chinese Medicine, College of Medicine, and Chinese Herbal Medicine Research Team, Healthy Aging Research Center, Chang Gung University, Taoyuan 333, Taiwan; E-Mail: htl@mail.cgu.edu.tw; 3 Department of Cosmetic Science and Research Center for Industry of Human Ecology, Chang Gung University of Science and Technology, Taoyuan 333, Taiwan; 4 National Museum of Marine Biology and Aquarium, Pingtung 944, Taiwan; E-Mails: pjsung@nmmba.gov.tw (P.-J.S.); x2219@nmmba.gov.tw (J.-H.S.); 5 Institute of Oceanography, National Taiwan University, Taipei 112, Taiwan; E-Mail: corallab@ntu.edu.tw; 6 Department of Life Science and Institute of Biotechnology and Graduate Institute of Marine Biotechnology, National Dong Hwa University, Pingtung 944, Taiwan; 7 Frontier Center for Ocean Science and Technology, National Sun Yat-sen University, Kaohsiung 804, Taiwan; 8 Graduate Institute of Natural Products, Kaohsiung Medical University, Kaohsiung 807, Taiwan; 9 Department of Medical Research, China Medical University Hospital, China Medical University, Taichung 404, Taiwan; 10 Doctoral Degree Program in Marine Biotechnology, National Sun Yat-sen University and Academia Sinica, Kaohsiung 804, Taiwan

**Keywords:** soft coral, *Cladiella hirsuta*, eunicellins, cytotoxic activity, anti-inflammatory activity

## Abstract

Four new eunicellin-type hirsutalins S–V (**1**–**4**), along with a known compound (–)-6α-hydroxy polyanthellin A (**5**), were isolated from the soft coral *Cladiella hirsuta*. The structures of the metabolites were determined by extensive spectroscopic analysis. Cytotoxity of compounds **1**–**5** against the proliferation of a limited panel of cancer cell lines was measured. Anti-inflammatory activity of compounds **1**–**5** was evaluated by measuring their ability in suppressing superoxide anion generation and elastase release in fMLP/CB-induced human neutrophils.

## 1. Introduction

**Chart 1:**
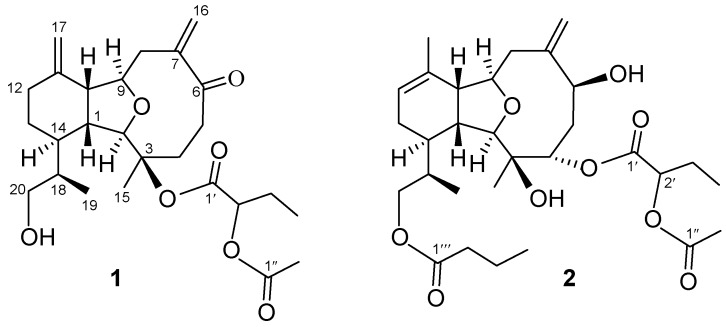

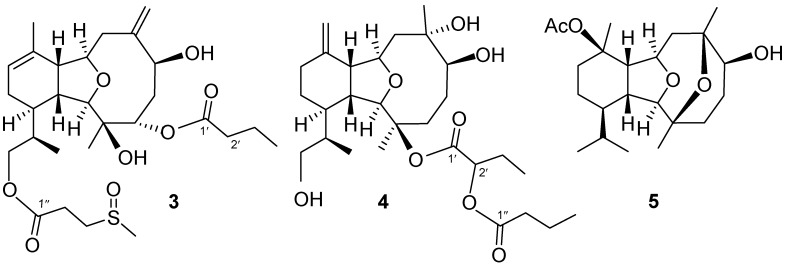
Structures of metabolites **1**–**5**.

The chemical investigations on soft corals of the genus *Cladiella* and *Klyxum* [1,2,3,4,5,6,7,8,9,10,11,12,13,14,15,16,17,18,19,20,21,22,23,24,25,26,27,28,29,30,31,32] have afforded series of eunicellin-based diterpenoids, of which many have been shown to exhibit attracting biological activities [8,10,11,12,13,14,15,16,17,18,19,20,21,22,23,24,25,26,27,28,29,30,31,32]. We have previously isolated some bioactive eunicellins and steroids from a Taiwanese soft coral *Cladiella hirsuta*. Our recent studies of *C. hirsuta* haveled to the discovery of 18 eunicellin-based diterpenoids, hirsutalins A–R [29,30,31], some of which have been found to possess cytotoxic [29,31] and anti-inflammatory activities [29,30,31]. In this paper, we further report the isolation of four new eunicellin-based compounds, hirsutalins S–V along with a known compound (–)-6α-hydroxy polyanthellin A (**5**) [32] from *C. hirsuta* (Chart 1). The structures of new compounds were determined by extensive spectroscopic analysis. Cytotoxicity of **1**–**5** against a limited panel of cancer cell lines and their anti-inflammatory activity, determined by their ability to inhibit the generation of super oxide anion and elastase release in *N*-formyl-methionyl-leucyl-phenylalanine/cytochalasin B (fMLP/CB)-induced human neutrophils, were studied in order to discover bioactive compounds from marine environment.

## 2. Results and Discussion

**Figure 1:**
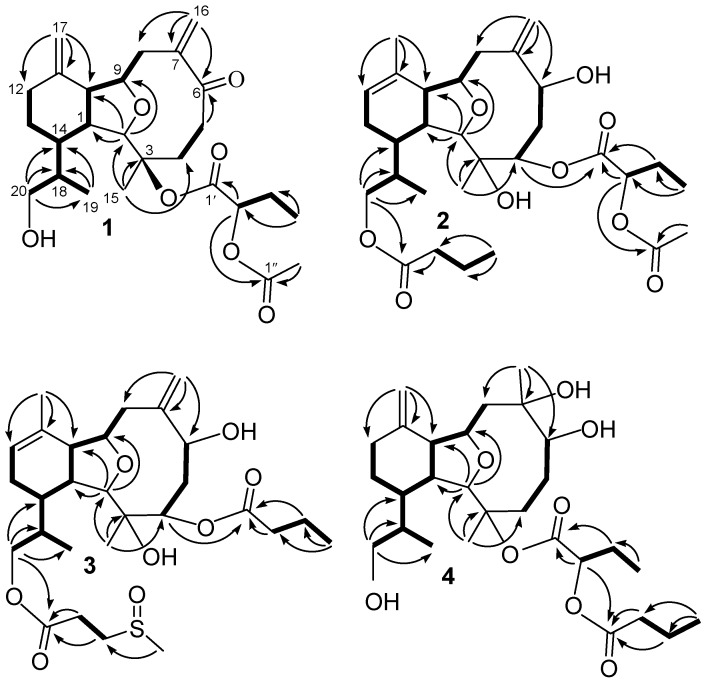
COSY and HMBC correlations for **1**–**4**.

Hirsutalin S (**1**) was isolated as a colorless oil. The HRESIMS (*m*/*z* 485.2512) of **1** established a molecular formula of C_26_H_38_O_7_. The IR spectrum of **1** showed the presence of hydroxy and carbonyl groups from absorptions at 3463 and 1740 cm^−1^, respectively. The ^1^H and ^13^C NMR data of **1** (Table 1) were found to be closely resembled to those of known metabolite hirsutalin R [32]. The only difference was the presence of 2-acetoxybutanoate (δ_C_ 169.0 (C), 73.9 (CH), 24.5 (CH_2_), and 9.7 (CH_3_); 171.0 (C) and 20.6 (CH_3_)) in **1**, instead of 2-butyryloxy butanoate at C-3 of hirsutalin R [32]. This was supported by the HMBC interaction of H-2′′ (δ 2.16) with carbonyl carbon resonating at δ 171.0. Moreover, the ^13^C NMR spectroscopic data (Table 1) of **1** showed the presence of two 1,1-disubstituted carbon-carbon double bonds (δ_C_ 147.6 (C) and 118.3 (CH_2_); 145.2 (C) and 111.6 (CH_2_)). The molecular framework of **1** was established by the complete analysis of its COSY and HMBC correlations (Figure 1). In the NOESY spectrum of **1**, the correlations between H-10 with H-1; H-1 with H_3_-19, suggested that H-1, H-10 and H_3_-19 are β-oriented. Besides, correlations of H-2 with H_3_-15 and H-14; H-9 with H-14, suggested that H-2, H-9, H-14 and H_3_-15 are α-oriented. Furthermore, the asymmetric center at C-18 was suggested to be *R*-configured on the basis of NOE correlations between the β-oriented H-1 and H_3_-19 and between the α-oriented H-2 and H-18. As the absolute configuration of hirsutalin A [29] and that of hirsutalin J except C-2′ configuration [30] have been completely assigned based on Mosher’s method, thus, the absolute configuration of **1**, except that of C-2′, should be revealed as depicted.

**Table 1:** NMR spectroscopic data for hirsutalins S (**1**) and T (**2**).

	1		2	
Position	δ_C_^a,b^	δ_H_ (*J* in Hz) ^c^	δ_C_^b,d^	δ_H_ (*J* in Hz) ^e^
1	45.1, CH ^b^	2.24, m	39.8; 39.7,^f^ CH	2.69, m
2	90.7, CH	3.70, s	87.64; 87.61, CH	3.86, d (6.0)
3	86.0, C		74.3, C	
4	32.4, CH_2_	2.13, m;	74.2, CH	5.08, dd (8.5, 3.5)
5	37.2, CH_2_	2.84, t (10.4); 2.35, m	37.9; 37.8, CH_2_	2.90, dq (15.5, 5.0); 1.78, m
6	206.5, C		72.6, CH	4.23, br s
7	147.6, C		147.7, C	
8	37.2, CH_2_	3.22, dd (13.2, 5.2); 2.40, m	40.1, CH_2_	2.42, m; 2.34, m
9	78.3, CH	4.07, m	81.80; 81.76, CH	4.15, m
10	48.7, CH	3.07, dd (9.6, 7.6)	44.5, CH	2.69, m
11	145.2, C		132.1; 132.0, C	
12	31.1, CH_2_	2.30, m; 2.11, m	122.02; 121.97, CH	5.46, s
13	25.8, CH_2_	1.68, m; 1.13, m	22.8; 22.8, CH_2_	2.09, m; 1.91, m
14	37.3, CH	1.68, m	34.4; 34.3, CH	1.84, m
15	22.7, CH_3_	1.48, s	22.6, CH_3_	1.38, s
16	118.3, CH_2_	5.62, s; 5.27, s	115.9; 115.8, CH_2_	5.62, s; 5.24, s
17	111.6, CH_2_	4.85, s; 4.72, s	22.3; 22.2, CH_3_	1.70, s
18	36.4, CH	1.79, m	33.8; 33.7, CH	1.84, m
19	16.3, CH_3_	1.03, d (7.2)	14.5; 14.3, CH_3_	0.86, d (6.5)
20	66.4, CH_2_	3.52, d (7.2)	67.8; 67.6, CH_2_	4.11, dd (9.5, 4.0); 3.89, m
1′	169.0, C		171.4;171.2, C	
2′	73.9, CH	4.76, t (6.8)	74.1, CH	4.84, dd (13.0, 6.0)
3′	24.5, CH_2_	1.87, m	24.4; 24.3, CH_2_	1.90, m
4′	9.7, CH_3_	1.03, t (7.6)	9.4; 9.3, CH_3_	1.03, t (7.5)
1′′	171.0, C		171.1; 171.0, C	
2′′	20.6, CH_3_	2.16, s	20.9; 20.5, CH_3_	2.13, s; 2.02, s
20-OCOPr			173.9; 173.7, C	
			36.2; 35.7, CH_2_	2.27, m;
			18.5; 18.3, CH_2_	1.64, m;
			13.7; 13.6, CH_3_	0.96, t (7.5); 0.94, t (7.5)

^a^ Spectra recorded at 100 MHz in CDCl_3_; ^b^ Attached protons were deduced by DEPT experiments; ^c^ Spectra recorded at 400 MHz in CDCl_3_; ^d^ Spectra recorded at 125 MHz in CDCl_3_; ^e^ Spectra recorded at 500 MHz in CDCl_3_; ^f^ Paired signals due to C-2′ epimeric mixture.

**Figure 2:**
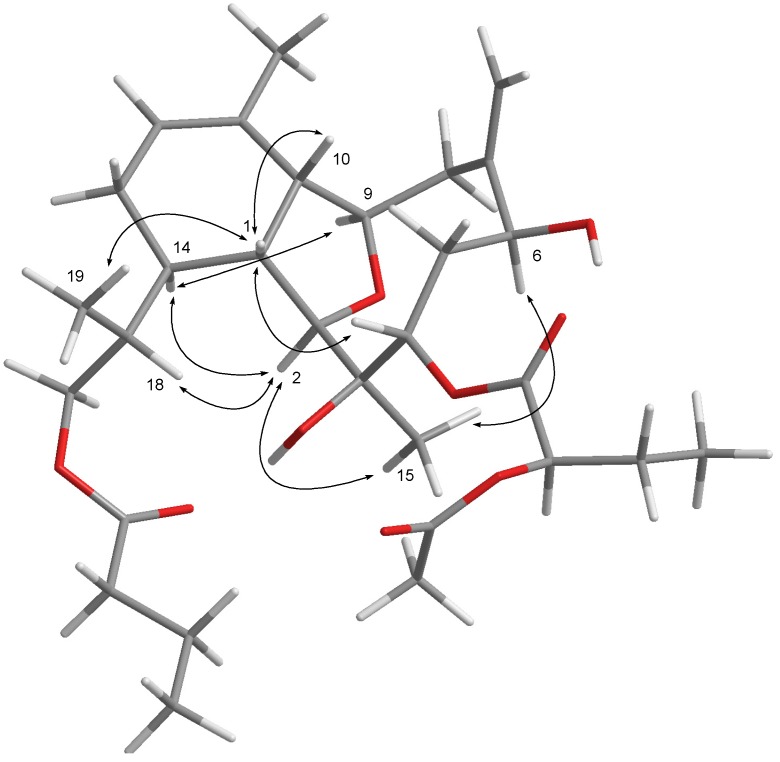
Key NOESY correlations for **2**.

Hirsutalin T (**2**) was also afforded as a colorless oil. Its molecular formula C_30_H_46_O_9_, was determined by HRESIMS (*m*/*z* 573.3036). The ^13^C NMR spectrum (Table 1) showed the presence of the 2-acetoxybutanoate (δ_C_ 171.2 (C), 74.1 (CH), 24.3 (CH_2_), and 9.3 (CH_3_); 171.0 (C) and 20.5 (CH_3_)) [29] and an *n*-butyrate (δ_C_173.9 (C), 36.2 (CH_2_), 18.5 (CH_2_), and 13.7 (CH_3_)). Comparison of the NMR data of **2** with those of the known compound hirsutalin A [29], it was found that a 2-hydroxybutyrate at C-3 and a methylene proton at C-4 in hirsutalin A were replaced by a hydroxy group and 2-acetoxybutanoate in **2**, respectively. This was confirmed by the downfield shift of C-3 (δ_C_ 86.9) of hirsutalin A, relative to that of **2** (δ_C_ 74.3), and the HMBC connectivity from H-4 (δ 5.08) to the carbonyl carbon resonating at δ171.2 (C) (Table 1). The structure of **2** was unambiguously determined by the extensive analysis of ^1^H–^1^H COSY and HMBC (Figure 1), and NOESY correlations (Figure 2). Moreover, compound **2** was obtained as a C-2′ epimeric mixture with a ratio of about 1:1 reflected by a pair of signals in the ^13^C NMR spectrum. Experiments were tried to separate an individual epimer but they were all unsuccessful.

The new eunicellin, hirsutalin U (**3**), gave the molecular formula C_28_H_44_O_8_S, on the basis of HRESIMS data (*m*/*z* 563.2657). NMR spectroscopic data of **3** (Table 2) showed the presence of the 3-methylsulfoxylpropionate substituent (δ_C_ 171.8 (C), 48.92 (CH_2_), 27.1 (CH_2_), and 38.6 (CH_3_)) [13] and an *n*-butyrate (δ_C_ 175.4 (C), 36.5 (CH_2_), 18.5 (CH_2_), and 13.7 (CH_3_)). The spectroscopic data (IR, ^1^H NMR, and ^13^C NMR) of **3** have similar structural features as those of a known one, hirsutalin J [30], except for the 2-butyryloxybutanoate at C-4 and the hydroxy group at C-20 in hirsutalin J were replaced by a *n*-butyrate group and 3-methylsulfoxylpropionate substituent in **3**, respectively. A paired methyl singlets at δ 2.58/2.59 in an approximate 1:1 ratio in the ^1^H NMR spectrum, and the doubling of signals of above methyl group with nearly the equal intensities in ^13^C NMR spectrum were observed, suggested the occurrence of nearly equal quantities of *R* and *S*-configured sulfoxide moiety (Table 2). Thus, compound **3** is possibly to be an artifact arisen from the oxidation of its sulfide precursor. The analysis of NOE correlations of **3** revealed the same relative configuration at C-1, C-2, C-3, C-4 C-6, C-9, C-10, C-14 and C-18 as that of **2**. The similar ^1^H NMR, COSY, HMBC correlations (Figure 1) and the analysis of NOE correlations of **3** further revealed the same relative configuration of both compounds. Thus, the structure of **3** was established.

**Table 2:** NMR spectroscopic data for hirsutalins U and V (**3** and **4**).

	3		4	
Position	δ_C_^a,b^	δ_H_ (*J* in Hz) ^c^	δ_C_^a,b^	δ_H_ (*J* in Hz) ^c^
1	39.8, CH ^b^	2.66, m	45.2, CH	2.12, m
2	87.3, CH	3.85, s	91.8, CH	3.61, s
3	74.3, C		87.9, C	
4	73.4, CH	4.93, m;	36.6, CH_2_	2.64, dd (14.8, 8.4); 1.86, m
5	37.7, CH_2_	3.01, m; 1.77, m	29.9, CH_2_	1.66, m; 1.56, m
6	72.7, CH	4.17, m	80.6, CH	4.58, d (6.8)
7	148.1, C		77.0, C	
8	40.0, CH_2_	2.35, m	45.5, CH_2_	2.02, m; 1.84, m
9	81.3, CH	4.19, m	78.4, CH	4.17, m
10	44.5, CH	2.66, m	53.8, CH	3.02, t (7.2)
11	132.3, C		147.1 , C	
12	121.4, CH	5.46, s	31.3 CH_2_	2.28, br d (13.2); 2.08, m
13	22.8, CH_2_	2.09, m; 1.91, m	25.3, CH_2_	1.64, m; 1.09, m
14	33.6, CH	1.84, m	38.4, CH	1.58, m
15	27.7, CH_3_	1.42, s	23.0, CH_3_	1.38, s
16	115.3, CH_2_	5.61, s; 5.22, s	22.4, CH_2_	1.25, s
17	22.1, CH_3_	1.68, s	109.8, CH_2_	4.71, s; 4.68, s
18	34.0, CH	1.82, m	37.5, CH	1.75, m
19	15.4, CH_3_	0.90, d (7.2)	10.6, CH_3_	0.80, d (6.8)
20	68.5, CH_2_	4.16, m; 4.05, m	66.5, CH_2_	3.53, d (6.8)
1′	175.4, C		169.1, C	
2′	36.5, CH_2_	2.41, m	74.0, CH	4.77, t (6.4)
3′	18.5, CH_2_	1.46, m	24.7, CH_2_	1.88, m
4′	13.7, CH_3_	0.97, t (7.2)	9.9, CH_3_	1.06, t (7.2)
1′′			173.5, C	
2′′			35.7, CH_2_	2.38, t (7.2)
3′′			18.3, CH_2_	1.69, m
4′′			13.6, CH_3_	0.97, t (7.2)
3-methylsulfoxylpropionate				
1′′	171.8; 171.3, C*^d^*			
2′′	48.92; 48.89, CH_2_	3.04, m; 2.88, m		
3′′	27.1; 26.7, CH_2_	2.83, m; 2.78, m		
4′′	38.6; 38.5, CH_3_	2.59, s; 2.58, s		

^a^ Spectra recorded at 100 MHz in CDCl_3_; ^b^ Attached protons deduced by DEPT experiments; ^c^ Spectra recorded at 400 MHz in CDCl_3_; ^d^ Paired signals of *R*/*S* stereoisomers at chiral sulfoxide.

**Figure 3:**
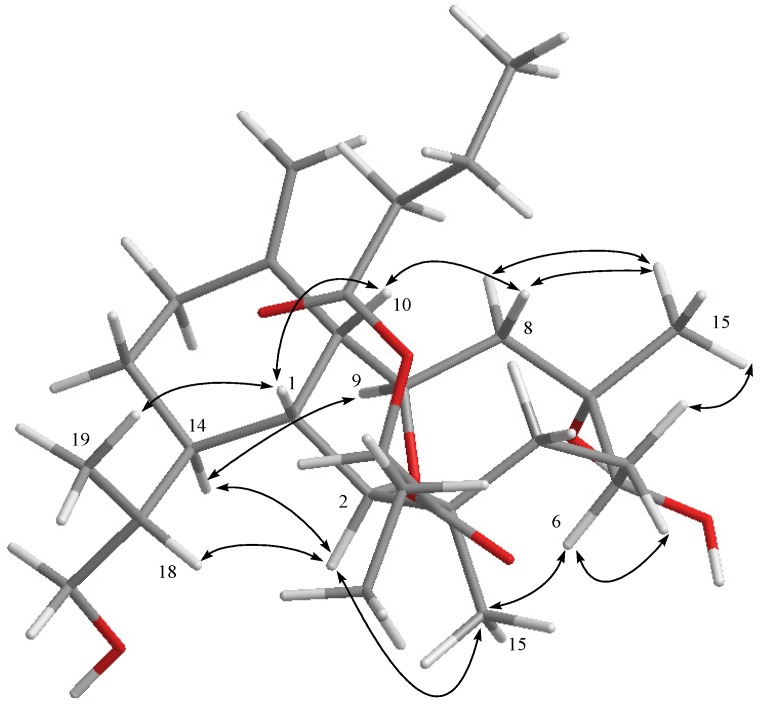
Key NOESY correlations for **4**.

Hirsutalin V (**4**) was obtained as a colorless oil with a molecular formula of C_2__8_H_46_O_8_. IR absorptions of **4** showed the presence of hydroxy and carbonyl groups at 3395 and 1738 cm^−^^1^, respectively. Two ester carbonyl carbons (δ_C_ 169.1 and 173.5) were correlated with the methine proton (H-2′, δ_H_ 4.77, t, *J* = 6.4 Hz) of a 2-butyryloxybutanoate unit in the HMBC spectrum. By comparison of the NMR data of **4** with those of hirsutalin C [29], it was found that a C-7/C-16 double bond in hirsutalin C was replaced by an oxymethine bearing a methyl and a hydroxy group in **4**, as confirmed by HMBC correlations observed from H_3_-16 (δ 1.25, 3H, s) to C-6 (δ 80.6, CH), C-7 (δ 77.0, C) and C-8 (δ 45.5, CH_2_). The planar structure of **4** was confirmed by careful analysis of COSY, HMBC, and NOESY correlations as shown in Figure 1 and Figure 3. Compounds **1**–**4** are likely in the same enantiomeric series as hirsutalin A and hirsutalin J, based on a shared biosynthetic pathway. Thus, these compounds were suggested to possess the absolute configurations as shown in structures **1**–**5**.

Cytotoxicity of compounds **1**–**5** against the proliferation of a limited panel of cancer cell lines, including P388 (murine leukemia), K562 (human erythro myeloblastoid leukemia), A549 (human lung adenocarcinoma), and HT-29 (human colon adenocarcinoma), was evaluated. However, none of the compounds showed any appreciable cytotoxicity at 20 μM. The *in vitro* pro-inflammatory of compounds **1**, **2**, and, **4** were evaluated by suppressing *N*-formyl-methionyl-leucyl-phenyl-alanine/cytochalasin B (fMLP/CB)-induced superoxide anion (O_2_^−•^) generation and elastase release in human neutrophils. As shown in Table 3, none of compounds showed significant reduction on the expression of superoxide anion generation, relative to the control cells stimulated with fMLP/CB at a concentration of 10 μg/mL. Further, compound **1** exhibited moderate inhibitory activity against elastase release (46.7% ± 8.0%), though it has shown poor superoxide anion generation (5.8% ± 0.8%) in the same fMLP/CB-stimulated cells at a concentration of 10 μg/mL.

**Table 3:** Effect of compounds **1**, **2**, and, **4** on superoxide anion generation and elastase release in *N*-formyl-methionyl-leucyl-phenylalanine/cytochalasin B (fMLP/CB)-induced human neutrophils at 10 μg/mL.

Compounds	Superoxide Anion	Elastase Release
	Inhibition %	Inhibition %
1	5.8 ± 0.8 **	46.7 ± 8.0 **
2	6.6 ± 3.4	19.3 ± 5.6 *
4	0.9 ± 2.6	4.8 ± 5.6

Percentage of inhibition (Inh %) at 10 μM concentration. Results are presented as mean ± S.E.M. (*n* = 3 or 4). * *p* < 0.05, ** *p* < 0.01 compared with the control value.

## 3. Experimental Section

### 3.1. General Experimental Procedures

Silica gel (230–400 mesh, Merck, Darmstadt, Germany) was used for column chromatography. Precoated silica gel plates (Merck, Kieselgel 60 F-254, 0.2 mm) were used for analytical TLC. High-performance liquid chromatography was performed on a Hitachi L-2455 HPLC apparatus (Hitachi Ltd., Tokyo, Japan) with a Supelco C_18_ column (250 × 21.2 mm, 5 μm). NMR spectra were recorded on a Varian UNITY INOVA-500 FT-NMR a Varian 400MR FT-NMR instrument (Varian Inc., Palo Alto, CA, USA) at 400 MHz for ^1^H and 100 MHz for ^13^C in CDCl_3_. LRMS and HRMS were obtained by ESI on a Bruker APEX II mass spectrometer (Bruker, Bremen, Germany). Optical rotations were measured on a JASCO P-1020 polarimeter. IR spectra were recorded on a JASCO FT/IR-4100 infrared spectrophotometer (Japan Spectroscopic Corporation, Tokyo, Japan).

### 3.2. Animal Material

The animal *Cladiella hirsuta* was collected by hand using SCUBA off the coast of Sianglu Islet (23°32′ N, 119°38′ E) in the region of Penghu Islands, in June 2008, at a depth of 10 m, and was stored in a freezer until extraction. A voucher sample (PI-20080610-17) was deposited at the Department of Marine Biotechnology and Resources, National Sun Yat-sen University.

### 3.3. Extraction and Separation

The frozen bodies of *C. hirsuta* (3.1 kg, wet wt) were sliced and exhaustively extracted with acetone (3 × 10 L).The organic extract was concentrated to an aqueous suspension and was partitioned between ethyl acetate (EtOAc) and H_2_O. The EtOAc layer was dried with anhydrous Na_2_SO_4_. After removal of solvent in vacuo, the residue (32.8 g) was subjected to column chromatography on silica gel and eluted with EtOAc in *n*-hexane (0%–100% of EtOAc, gradient) and further with MeOH in EtOAc of increasing polarity to yield 25 fractions. Fraction 18, eluting with *n*-hexane–EtOAc (1:1), was rechromatographed over a Sephadex LH-20 column using acetone as the mobile phase to afford four subfractions (A1–A4). Subfractions A3 and A4 were separated by reversed-phase HPLC (MeOH–H_2_O, 3:1 and 2:1) to afford compound **1** (5.8 mg). Fraction 19, eluting with *n*-hexane–EtOAc (1:2), was rechromatographed over a Sephadex LH-20 column, using acetone as the mobile phase, to afford four subfractions (B1–B4). Subfractions B2 and B3 were separated by reversed-phase HPLC (acetonitrile–H_2_O, 3:1 and 2:1) to afford compounds **2** (1.5 mg) and **5** (1.3 mg). Fraction 23, eluting with EtOAc (1:2), was rechromatographed over a Sephadex LH-20 column, using acetone as the mobile phase, to afford four subfractions (B1–B4). Subfractions B2 and B3 were separated by reversed-phase HPLC (acetonitrile–H_2_O, 1.5:1) to afford compounds **3** (2.6 mg) and **4** (1.5 mg).

Hirsutalin S (**1**): colorless oil; [α]^25^_D_ +66 (*c* 0.40, CHCl_3_); IR (neat) ν_max_ 3463 and 1740 cm^−1^;^13^C and ^1^H NMR data (400 MHz; CDCl_3_), see Table 1; ESIMS *m*/*z* 485 [M + Na]^+^; HRESIMS *m*/*z* 485.2512 [M + Na]^+^ (calcd for C_26_H_38_O_7_Na, 485.2515) (Supplementary Information, Figures S1–S3).

Hirsutalin T (**2**): colorless oil; [α]^25^_D_ +26.3 (*c* 0.43, CHCl_3_); IR (neat) ν_max_ 3452 and 1738 cm^−1^; ^13^C and ^1^H NMR data (500 MHz; CDCl_3_), see Table 1; ESIMS *m*/*z* 573 [M + Na]^+^; HRESIMS *m*/*z* 573.3036 [M + Na]^+^ (calcd for C_30_H_46_O_9_Na, 573.3039) (Supplementary Information, Figures S4–S6).

Hirsutalin U (**3**): colorless oil; [α]^25^_D_ +11 (*c* 0.74, CHCl_3_); IR (neat) ν_max_ 3442 and 1733 cm^−1^; ^13^C and ^1^H NMR data (400 MHz; CDCl_3_), see Table 2; ESIMS *m*/*z* 563 [M + Na]^+^; HRESIMS *m*/*z* 563.2657 [M + Na]^+^ (calcd for C_28_H_44_O_8_SNa, 563.2654) (Supplementary Information, Figures S7–S9).

Hirsutalin V (**4**): colorless oil; [α]^25^_D_ −18.1 (*c* 0.51, CHCl_3_); IR (neat) ν_max_ 3395 and 1738 cm^−1^; ^13^C and ^1^H NMR data (400 MHz; CDCl_3_), see Table 2; ESIMS *m*/*z* 533 [M + Na]^+^; HRESIMS *m*/*z* 533.3094 [M + Na]^+^ (calcd for C_28_H_46_O_8_Na, 533.3092) (Supplementary Information, Figures S10–S12).

### 3.4. Cytotoxicity Testing

Cell lines were purchased from the American Type Culture Collection (ATCC). Cytotoxicity assays of compounds **1**–**5** were performed using the Alamar Blue assay [33,34].

### 3.5. In Vitro Anti-Inflammatory Assay

Human neutrophils were obtained using dextran sedimentation and Ficoll centrifugation. Measurements of superoxide anion generation and elastase release were performed according to previously described procedures. [35,36]. LY294002, a phosphatidylinositol-3-kinase inhibitor, was used as a positive control for inhibition of superoxide anion generation and elastase release with percentage inhibitions of 96.1% ± 4.9% in 10 μg/mL and 97.9% ± 7.7% in 10 μg/mL, respectively.

## 4. Conclusions

Our investigation demonstrated that the soft coral, *C. hirsuta*, could be a good source of bioactive substances. It is worthwhile to mention that eunicellin-type metabolite containing a sulfoxide, compound **3**, was discovered for the first time from the soft coral *C. hirsuta*. Compound **1** was shown to display inhibitory activity against elastase release.

## Supplementary Files

Supplementary File 1
