# Supplementary material for: Eunicellin-Based Diterpenoids, Hirsutalins S–V, from the Formosan Soft Coral Cladiella hirsuta
Source: Mar Drugs. 2015 Apr 30;13(5):2757–69. doi: 10.3390/md13052757 (PMC4446604; doi:10.3390/md13052757)
Supplement: Supplementary File 1 [file marinedrugs-13-02757-s001.pdf]

Supplementary Information

List of Contents

Figure S1. HRESIMS spectrum of **1**.
Figure S2. ^{1}H NMR spectrum of **1** in $CDCl_3$.
Figure S3. ^{13}C NMR spectrum of **1** in $CDCl_3$.
Figure S4. HRESIMS spectrum of **2**.
Figure S5. ^{1}H NMR spectrum of **2** in $CDCl_3$.
Figure S6. ^{13}C NMR spectrum of **2** in $CDCl_3$.
Figure S7. HRESIMS spectrum of **3**.
Figure S8. ^{1}H NMR spectrum of **3** in $CDCl_3$.
Figure S9. ^{13}C NMR spectrum of **3** in $CDCl_3$.
Figure S10. HRESIMS spectrum of **4**.
Figure S11. ^{1}H NMR spectrum of **4** in $CDCl_3$.
Figure S12. ^{13}C NMR spectrum of **4** in $CDCl_3$.

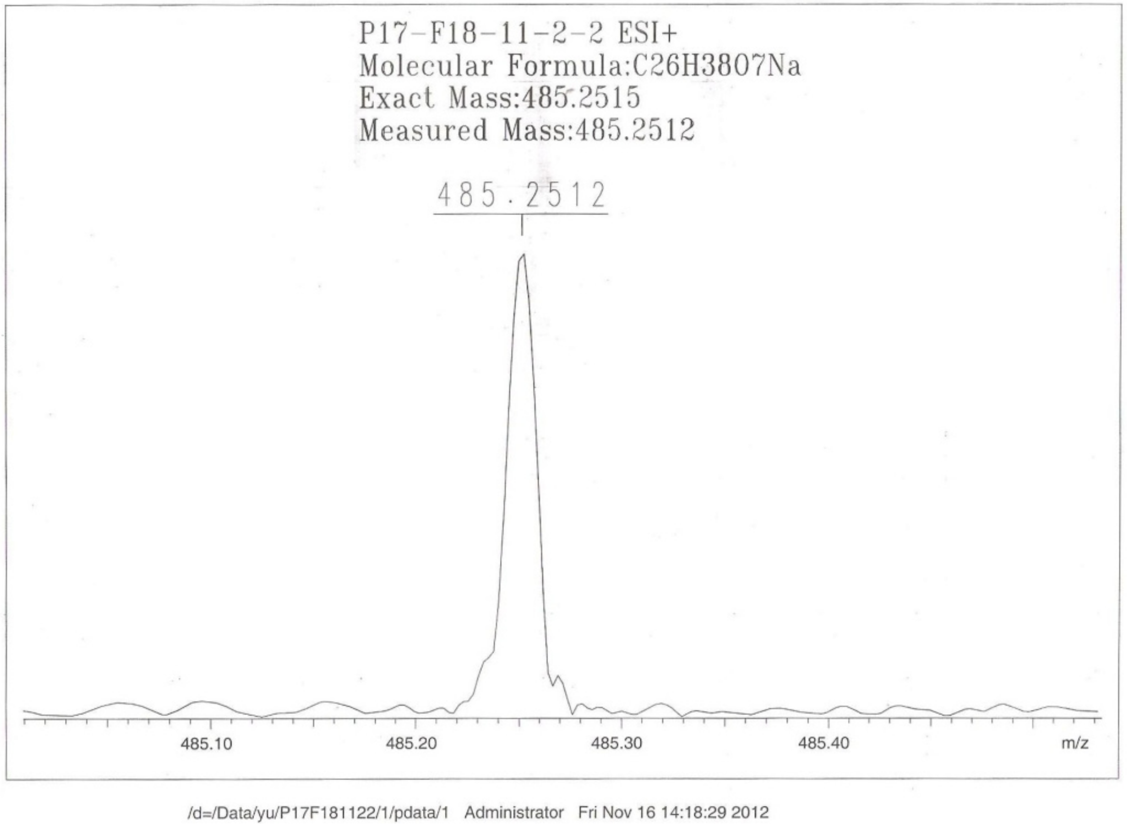

Figure S1. HRESIMS spectrum of **1**.

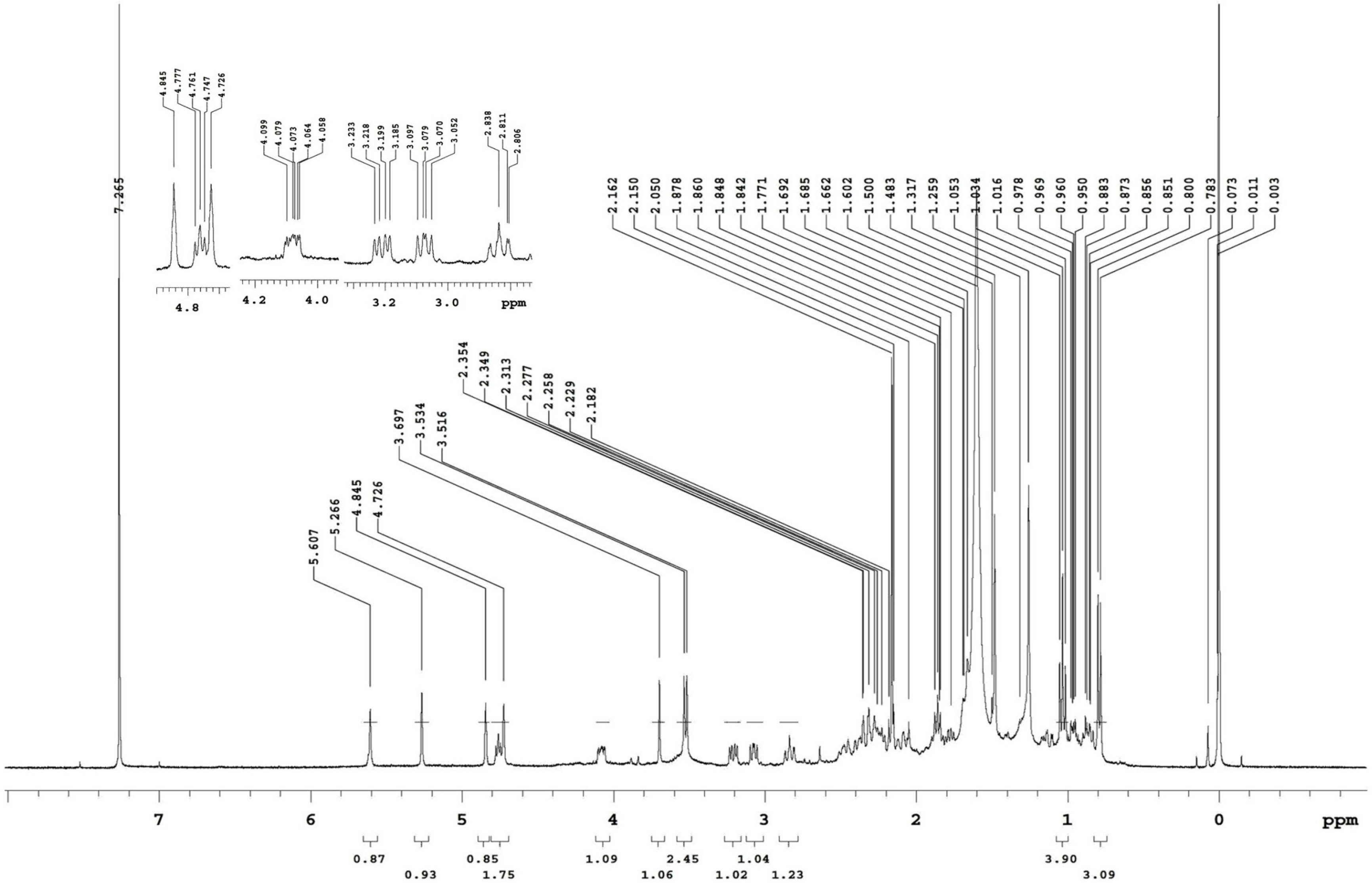

Figure S2. ^{1}H NMR spectrum of **1** in $CDCl_3$.

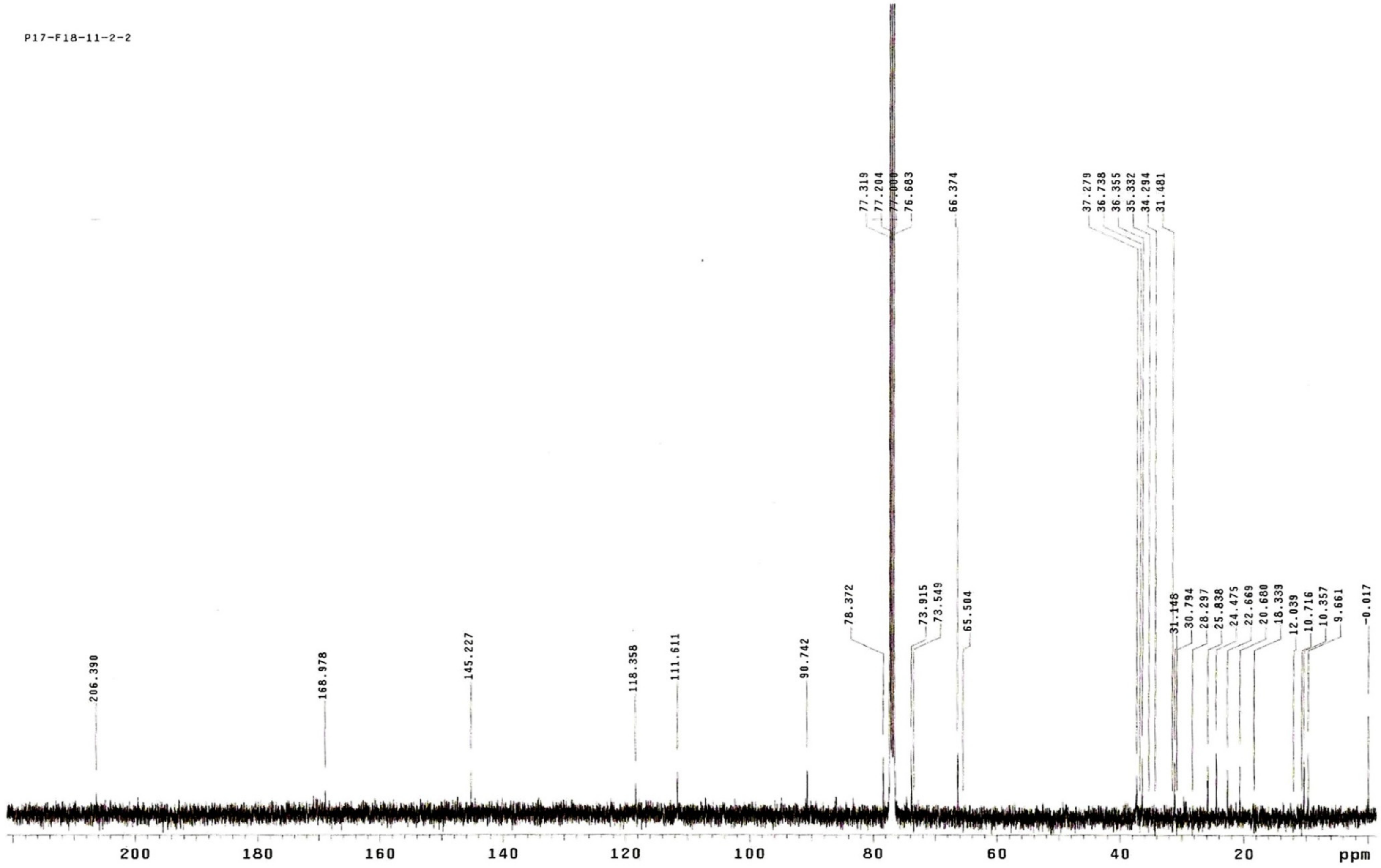

Figure S3. ^{13}C NMR spectrum of **1** in $CDCl_3$.

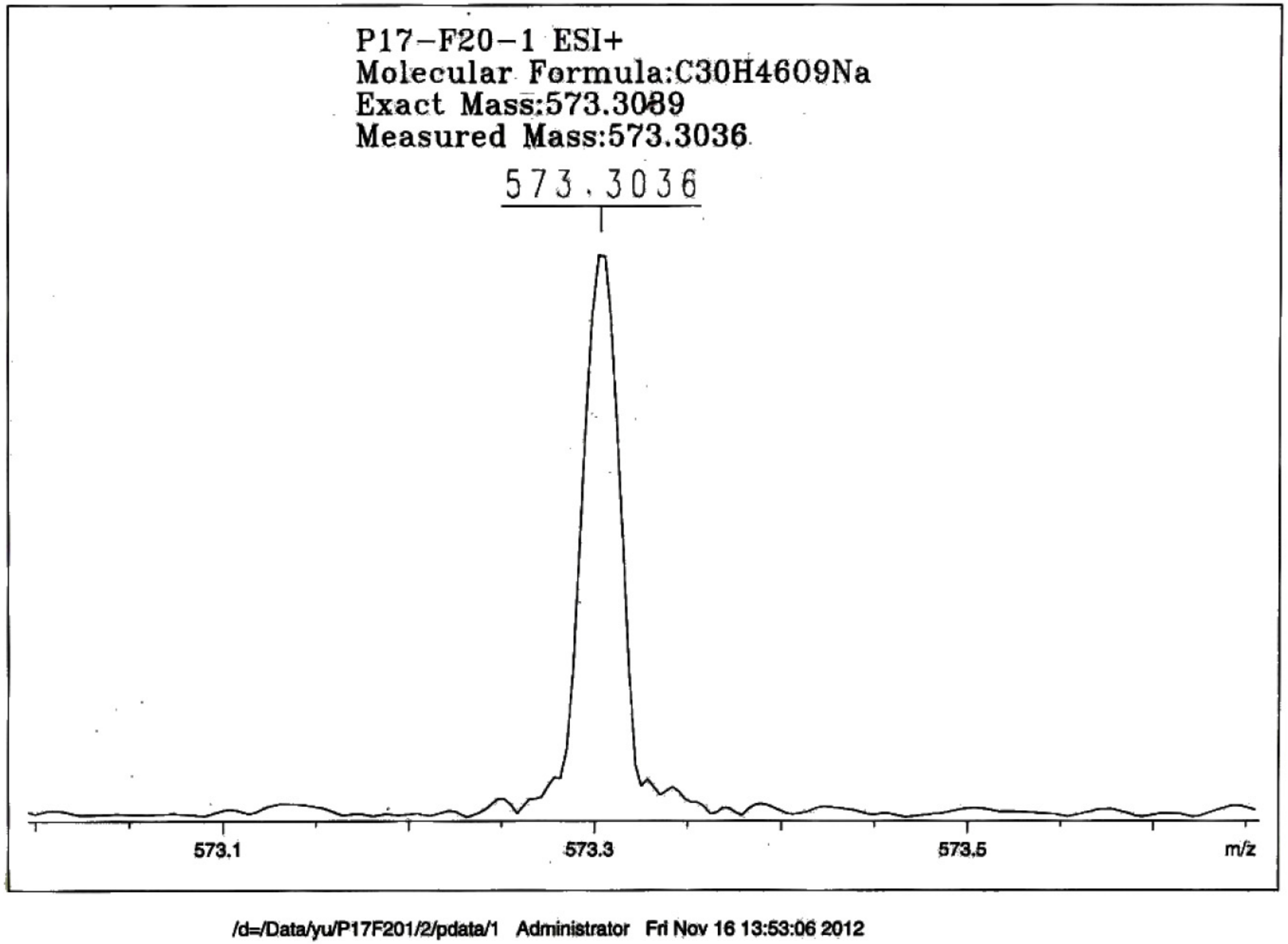

Figure S4. HRESIMS spectrum of **2**.

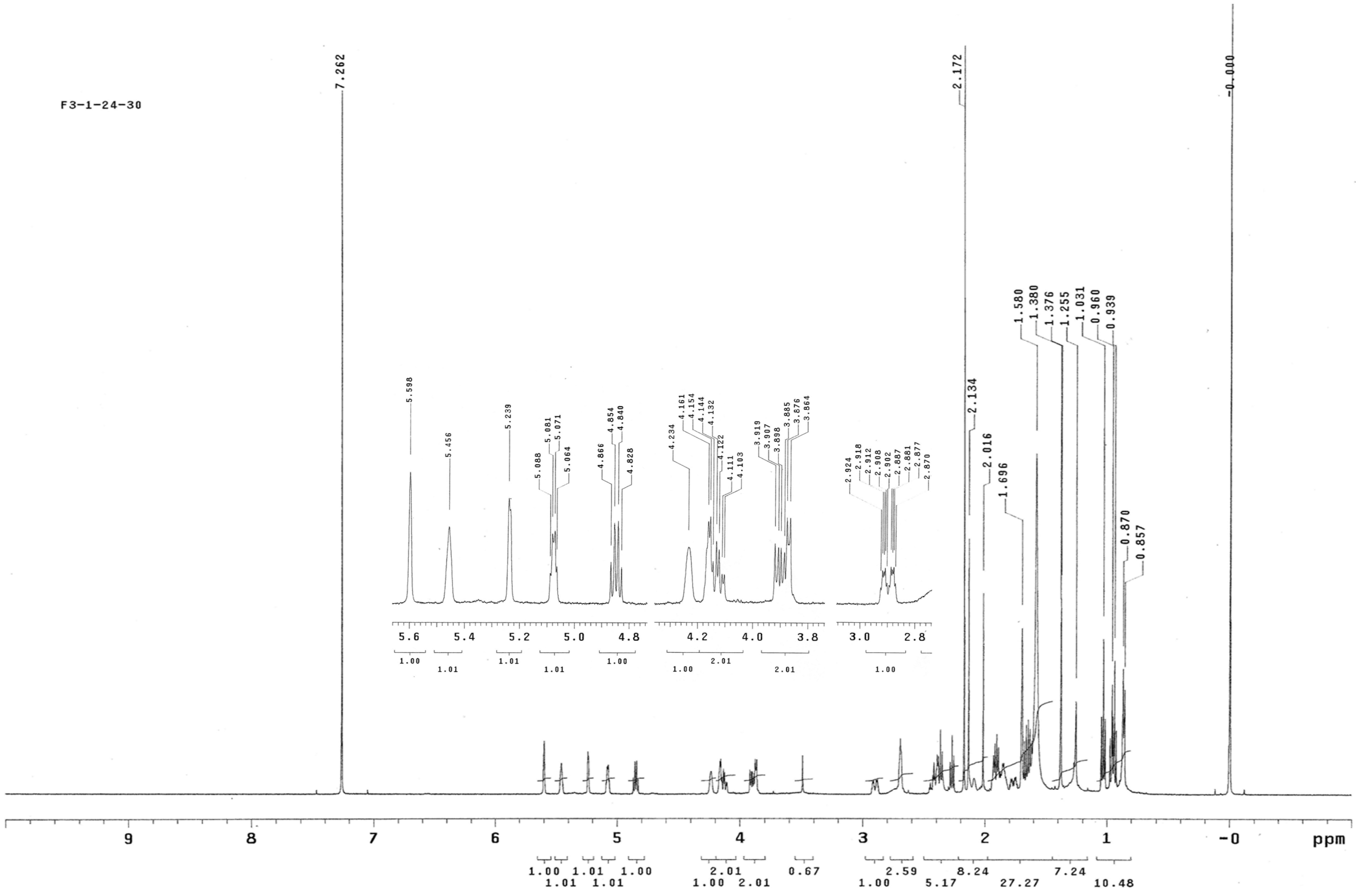

Figure S5. ^{1}H NMR spectrum of **2** in $CDCl_3$.

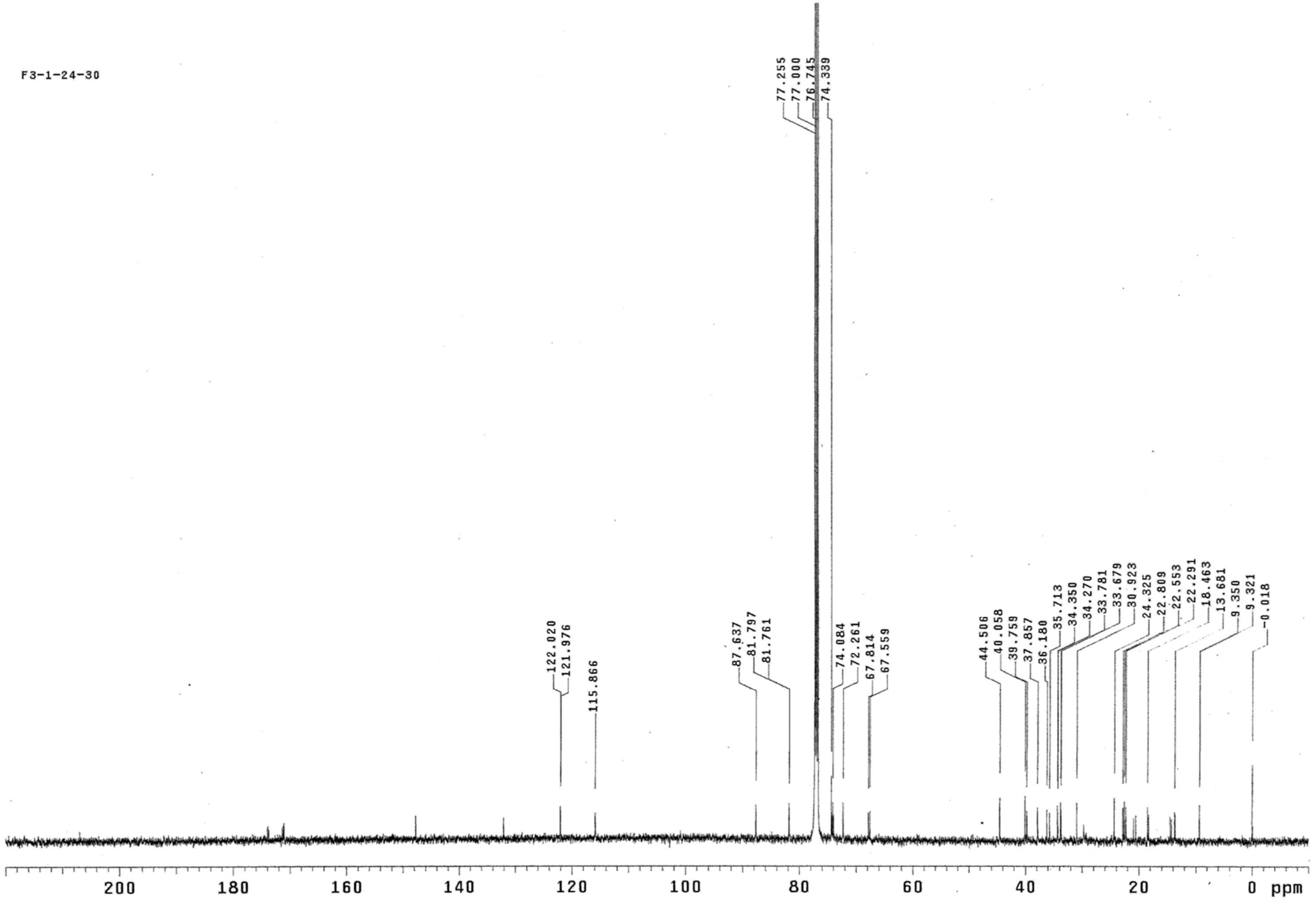

Figure S6. ^{13}C NMR spectrum of **2** in $CDCl_3$.

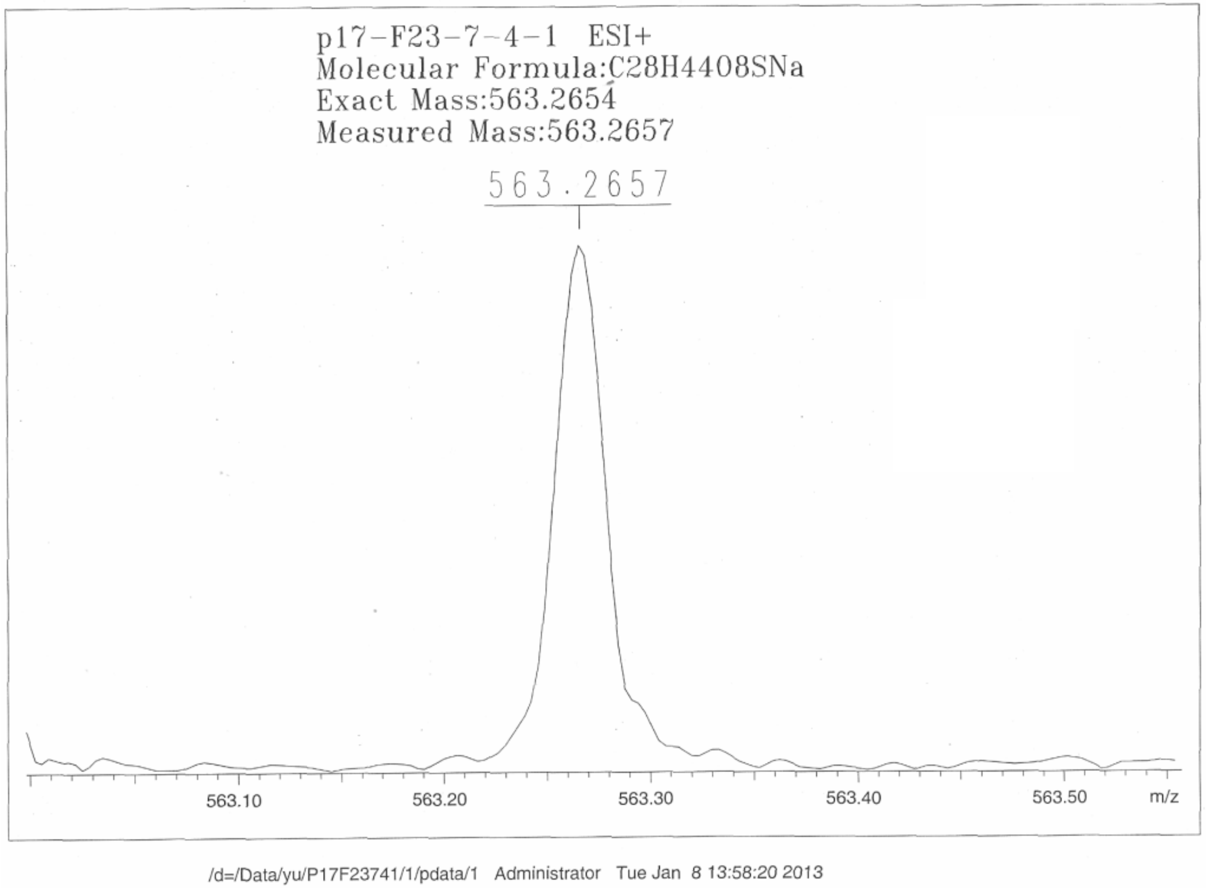

Figure S7. HRESIMS spectrum of **3**.

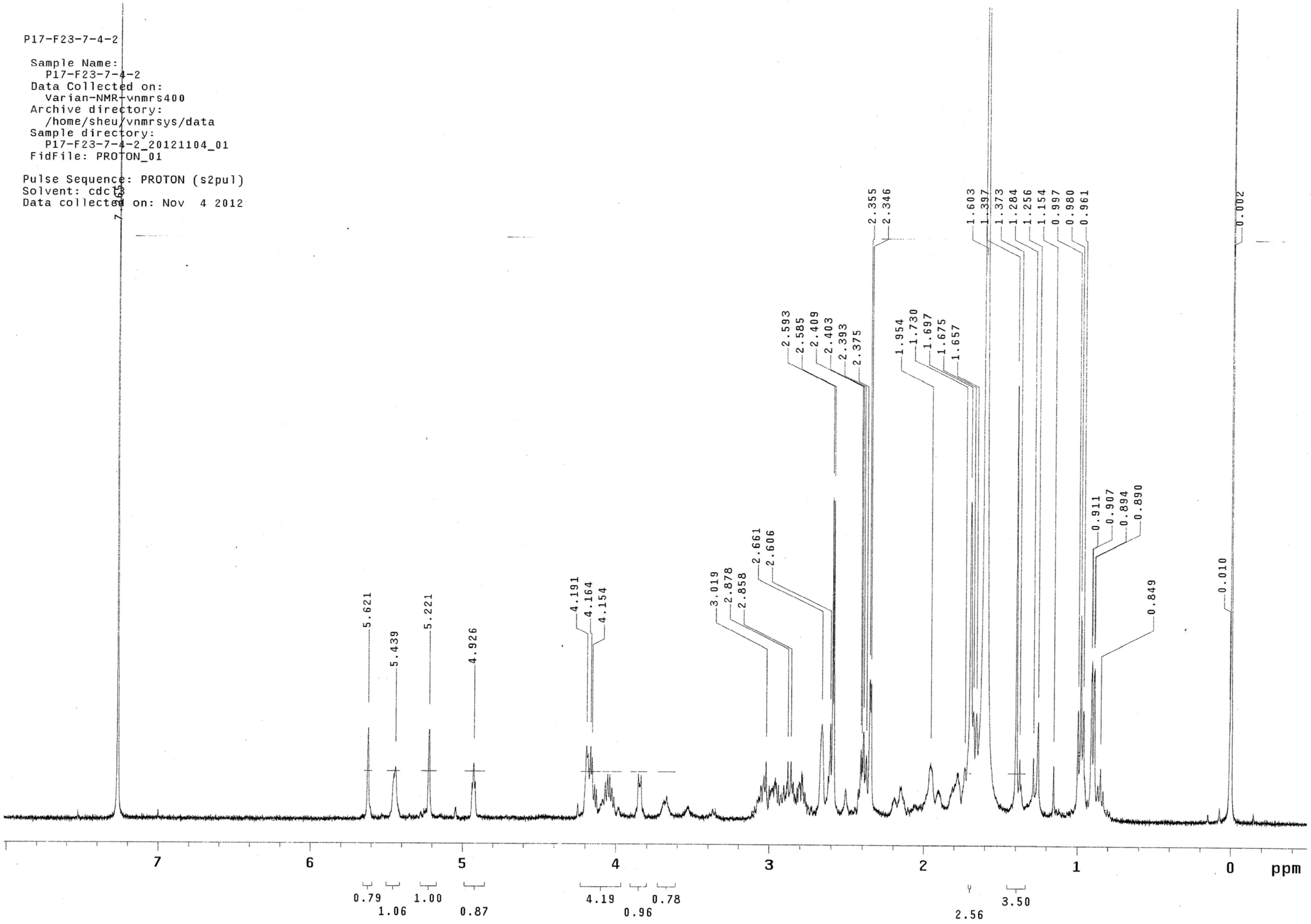

Figure S8. ^{1}H NMR spectrum of **3** in $CDCl_3$.

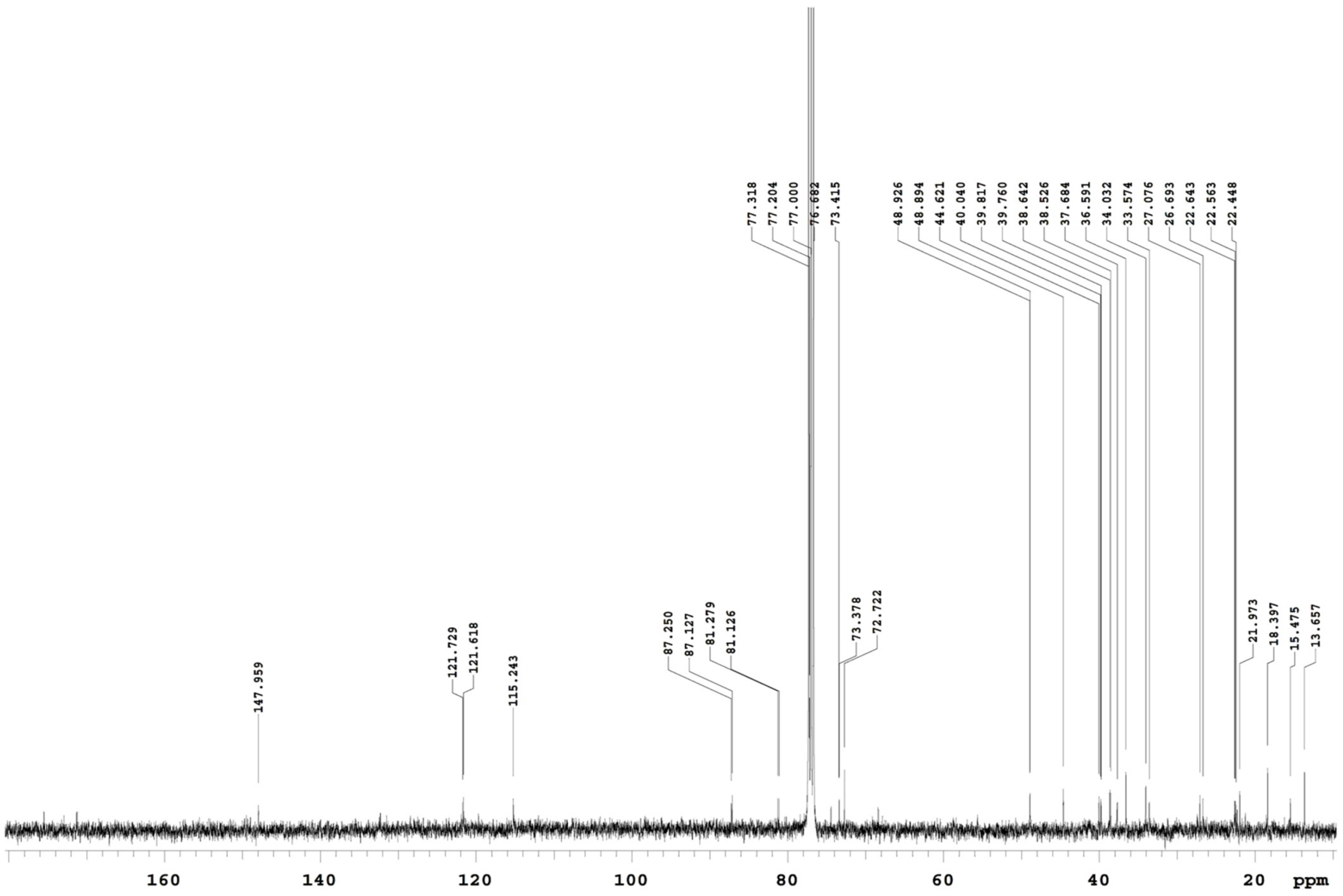

Figure S9. ^{13}C NMR spectrum of **3** in $CDCl_3$.

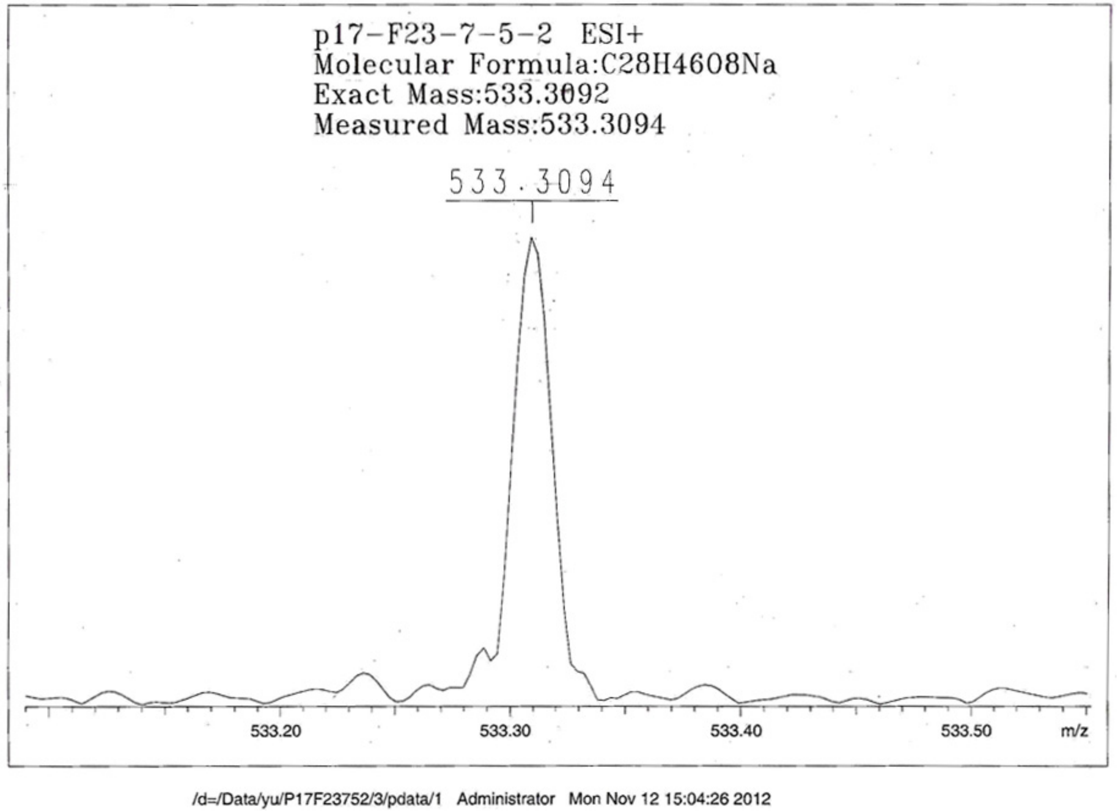

Figure S10. HRESIMS spectrum of **4**.

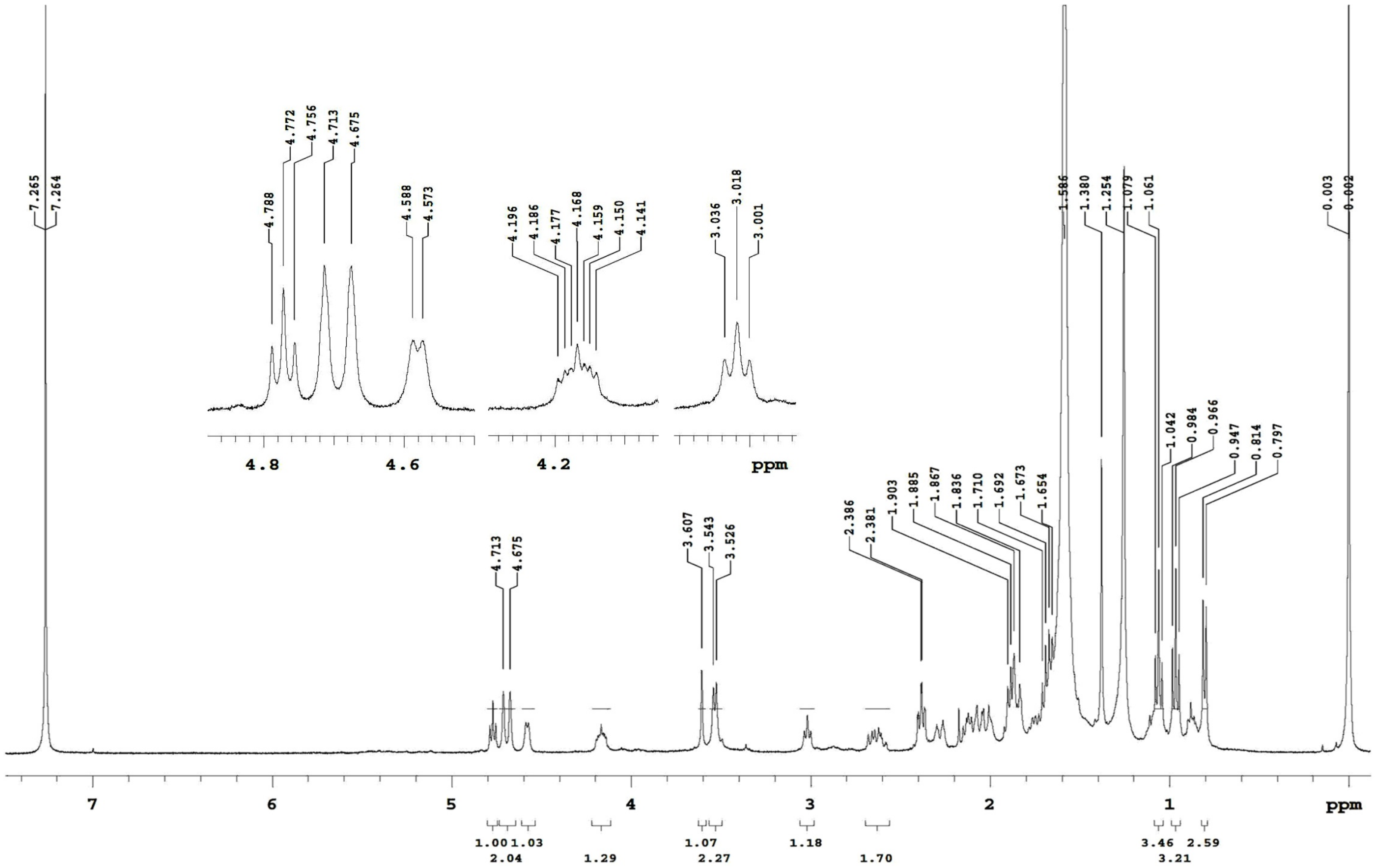

Figure S11. ^{1}H NMR spectrum of **4** in $CDCl_3$.

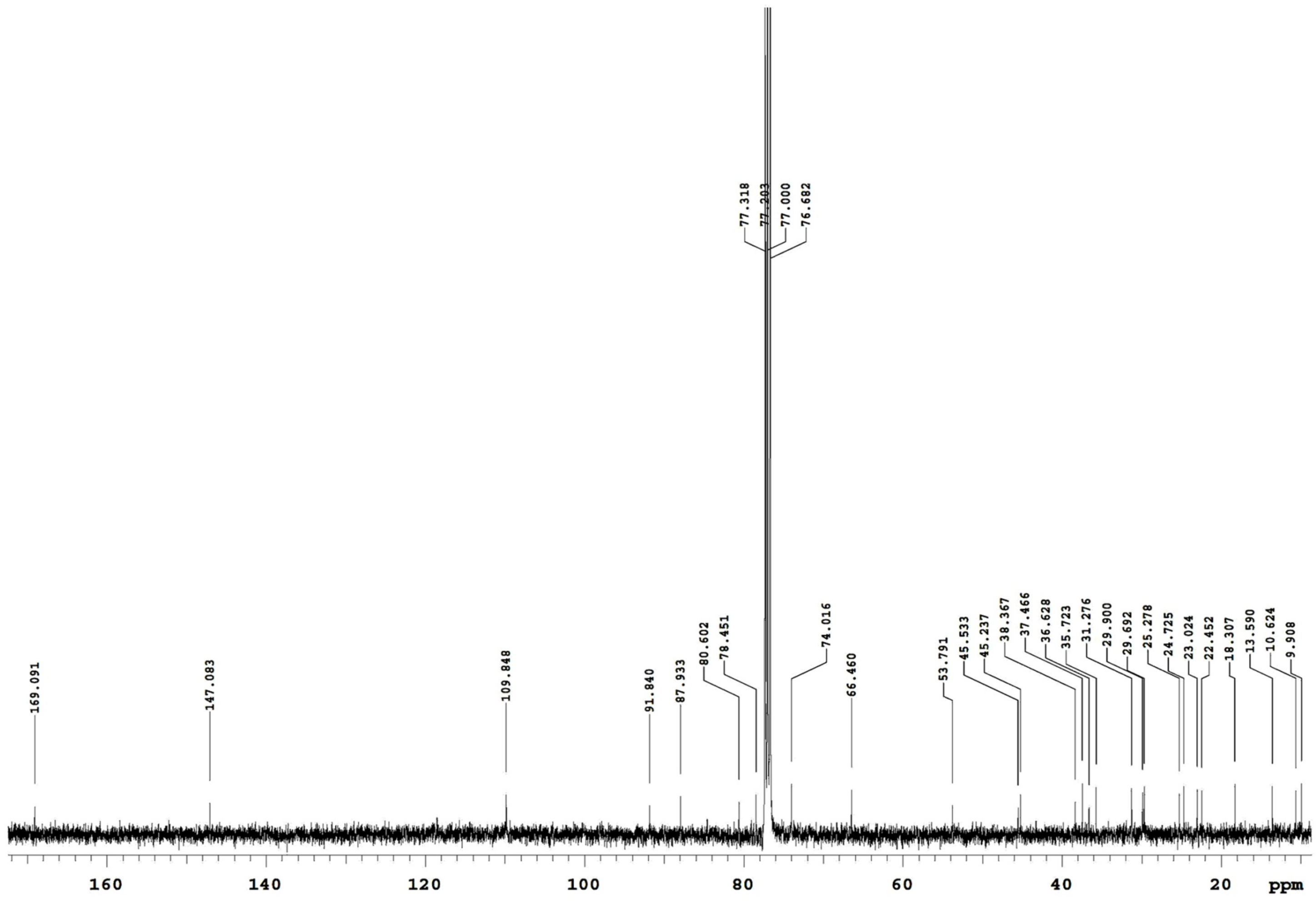

Figure S12. ^{13}C NMR spectrum of **4** in $CDCl_3$.

© 2015 by the authors; licensee MDPI, Basel, Switzerland. This article is an open access article distributed under the terms and conditions of the Creative Commons Attribution license (http://creativecommons.org/licenses/by/4.0/).
